# Design and fabrication of box-type passive solar dryer (BTPSD) with thermal insulation material for valorizing biomass and neutral lipids of marine *Chlorella vulgaris* for biodiesel application

**DOI:** 10.1038/s41598-022-09665-0

**Published:** 2022-04-11

**Authors:** N. Kalaiselvan, Thangavel Mathimani

**Affiliations:** Department of Energy and Environment, National Institute of Technology, Tiruchirappalli, Tamil Nadu 620015 India

**Keywords:** Industrial microbiology, Environmental sciences

## Abstract

The staggering rate of population growth has augmented the reliance on fossil fuel utilization, and it kindled the society to explore alternative and sustainable sources of energy. In this regard, biodiesel from microalgae came to the limelight; but crucial energy-consuming and expensive processes like cultivation, harvesting, and drying make the microalgal biodiesel unsustainable and economically unfeasible. To surpass these impediments, in this research work, a low-cost box-type passive solar dryer (BTPSD) is designed and fabricated with zero energy consumption mode and compared with conventional hot air oven for drying the biomass, neutral lipids of the marine microalga *Chlorella vulgaris* for biodiesel application. The onset of the work, BTPSD with 2 cm thickness of glass wool as TIM (thermal insulation material), 4 cm TIM thickness and no TIM was simulated for thermal storage behaviour using ANSYS FLUENT 19.2 Computational Fluid Dynamics tool and based on the results, 4 cm TIM thickness was chosen for experimentation. The time taken by BTPSD and hot air oven to remove the moisture from algal biomass is 3 and 2 h, respectively, whereas for neutral lipids drying, it was 4 and 3.5 h, respectively. Though there is a little difference in drying time, neutral lipid and FAME content from both drying systems are tantamount, i.e., ~ 12% neutral lipid and 95% FAME. Further, the percentage of vital fatty acids identified from BTPSD and hot air oven methods are almost similar, i.e., C16:0 (23.4%), C18:1 (14.3%), C18:3 (11.42%), C18:1 (9.22%). Though the time taken for valorizing biomass and neutral lipids of *C. vulgaris* by BTPSD is slightly longer than hot air oven, low energy consumption and cost-effectiveness make the BTPSD a promising system to scale down the microalgal biodiesel production cost significantly.

## Introduction

In developing countries, fossil fuel-based energy is being used dramatically for transportation, and excessive dependency on fossil fuel leads to increased carbon dioxide emissions. Irreversible impacts like climate change are expected if the current carbon dioxide emission increases from 385 ppmv (parts per million by volume)^1^. Therefore, the momentum has been shifted towards exploring renewable energies for sustainability. The utilization of renewable energy has increased rapidly in all sectors, especially in the transportation sector and electrical energy applications^2^. Of the renewable energy sources, biofuels from biomass have recently got rampant attention owing to their manifold benefits. Notably, biofuels produced from third-generation feedstock, i.e., microalgae came to the limelight with a positive intent to replace the existing conventional fuels^3,4^.

Nevertheless, to the best of our knowledge, microalgal biodiesel at an affordable cost or par with current diesel fuel price has not yet been produced. The primary constraints in producing commercially feasible/economically viable microalgal biodiesel are cultivation costs and expensive biomass processing methods. Cultivation and preservation of algal biomass is a paramount challenge to develop microalgal based alternative fuel for wide-range applications^5^. In addition, dewatering/drying of biomass and evaporation of solvents in extracted lipids using either hot air oven/lyophilization was identified to be expensive and energy-intensive processes. In this case, a cost-effective and less energy-consuming process can be an ideal choice to reduce the overall biodiesel production expenses. Energy consumed for drying microalgae is very high and it is estimated to be 84% of total energy consumption in algal biodiesel production^6^ and therefore, an appropriate drying method is essential to decrease the overall outlay of biodiesel production^7^. In this perspective, solar drying is an attractive option with zero-emission and meager energy consumption^8^. As one of the renewable energies, solar thermal technology is gaining importance as an energy-saving measure in drying and preservation application^9^. Solar technology is the most preferred renewable energy since it is an inexhaustible, abundant source with zero CO_2_ emission^10^. Solar thermal air heaters are compact devices to heat air by utilizing solar thermal energy, and they are being employed for many applications requiring low to moderate temperature (below 80 °C), such as crop drying and space heating^11^. Further, a solar dryer with thermal insulation material is advantageous as it absorbs excess energy during sunshine hours and discharges the same in poor sunlight to enhance the drying and many thermal insulation materials are being used in a solar dryer. There are numerous studies on using a direct or indirect solar dryer to remove the moisture from medicinal plants, edible products, and other agricultural products^12,13^. Only few studies were undertaken on solar drying of algal species like indirect solar dryer for *Spirulina platensis*^10^, open sun-light, and solar chimney drying for *Chlorella* sp.^14^, cabinet type solar dryer for the cyanobacterium-*Spirulina* sp. and microalga *Scenedesmus* sp.^15^, and open sun and solar drying of *Chlorella* sp.^7^.

Though solar dryer was reported in various literatures for drying agricultural products, valorization of biomass and neutral lipids of marine/hypersaline microalgal strains using passive/direct box-type solar dryer is not yet reported and quantity, quality of microalgal biochemical constituents especially lipids and fatty acids under solar dried and conventional hot air oven dried biomass have not been studied to the best of our knowledge. Considering the above issues in microalgal biodiesel production and the advantages of solar dryer, this present research is undertaken to evaluate the algal biomass and neutral lipid drying capability of the box-type passive solar dryer (henceforth referred to as BTPSD) and conventional hot air oven. A low-cost box type solar dryer is designed and fabricated with glass wool as thermal insulation material (TIM). Drying rate and moisture removed by the BTPSD was comparatively analyzed with conventional hot air oven system. The neutral lipids dried under hot air oven and BTPSD was subjected for FAME production, and its fatty acid composition and quality was examined for fuel characteristics.

## Results and discussion

**Figure 1:**
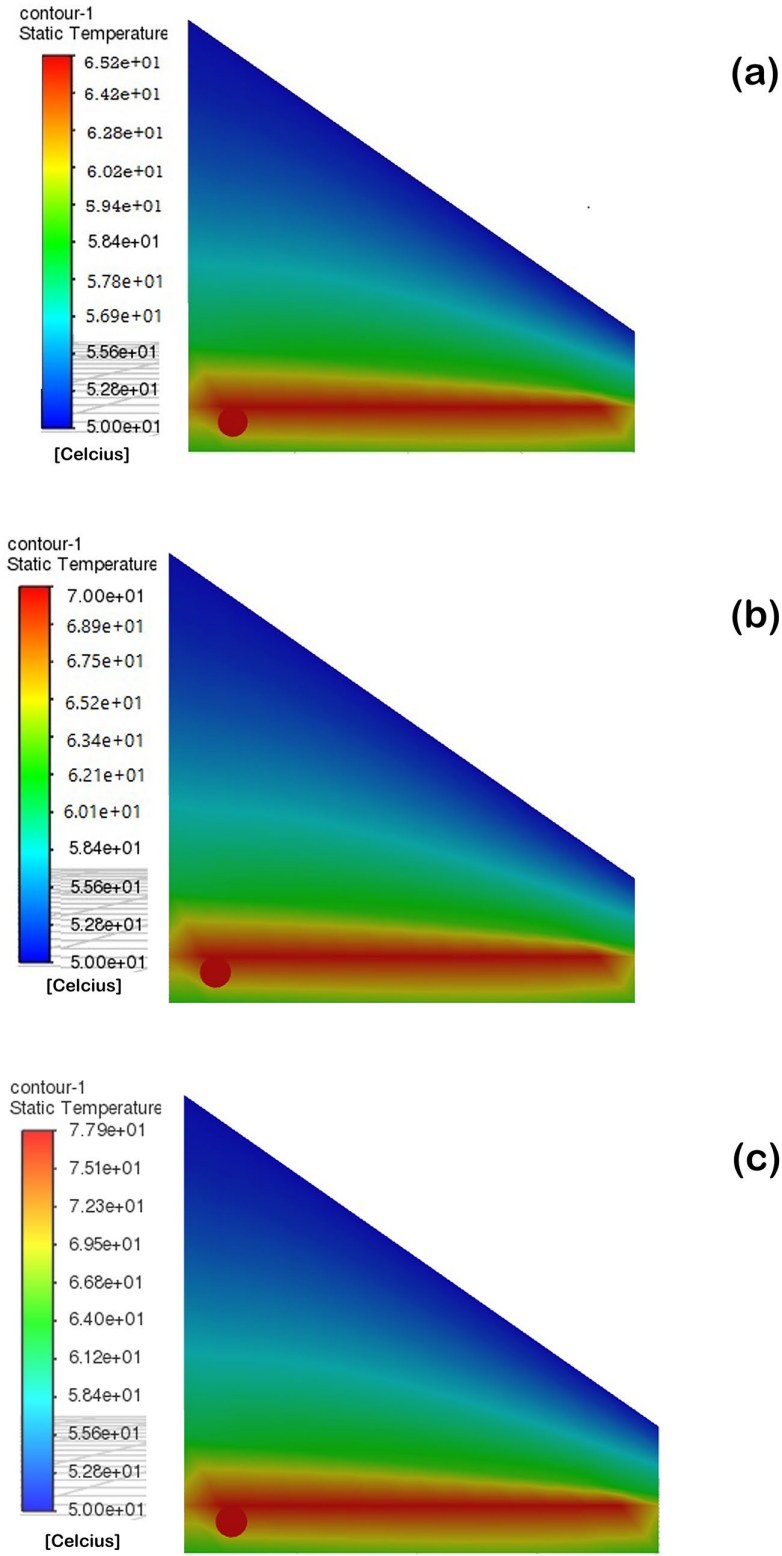
Simulation result of BTPSD with input temperature 50 °C (i) BTPSD without TIM (ii BTPSD 2 cm TIM thickness (iii) BTPSD with 4 cm TIM thickness.

**Figure 2:**
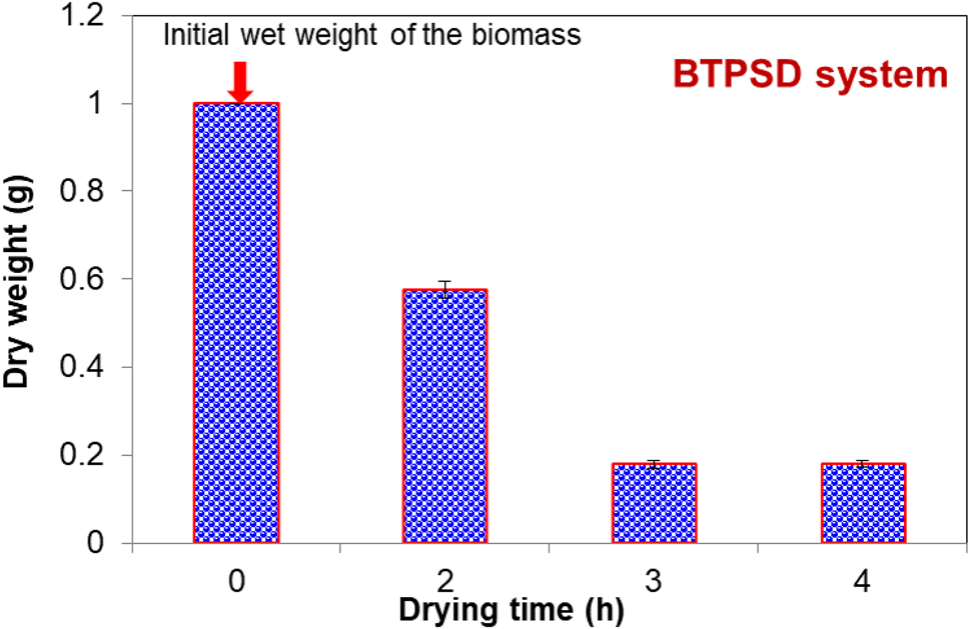
The biomass of the *C. vulgaris* strain dried under BTPSD with respect to drying time (h).

The significant steps in microalgal biodiesel production entail feedstock selection, cultivation, harvesting, drying/dewatering, oil extraction, and transesterification^16^. Among the steps, biomass drying is one of the imperative processes to remove the water content or moisture content present in the wet biomass. Though various modes of biomass drying have been proposed till now, a cost-effective and energy-efficient protocol is still under exploration. From this perspective, the present study has proposed box- type passive solar dryer for biomass and lipid drying in a cost-effective way. At the outset of the experimental work, the solar dryer with pertinent configurations needs to be chosen for efficient removal of moisture from algal biomass and also evaporating solvent from extracted lipids. Therefore, solar dryer with better thermal storage using different TIM thicknesses such as 2 cm and 4 cm were optimized initially using ANSYS software. The entire simulation was done with the ambient condition for three models; solar dryer with 2 cm TIM thickness, solar dryer with 4 cm TIM thickness and solar dryer without any TIM. For all three models, a similar temperature of 50 °C is fed as input to the top layer of the dryer. The simulation output of solar dryer with 2 and 4 cm TIM thickness and without TIM is shown in Fig. 1a–c. In the solar dryer with 4 cm TIM thickness, at 50 °C heat input, the absorber plate temperature varied from 74 to 77 °C, which is 4 °C higher than the solar dryer with 2 cm TIM thickness. For the solar dryer with 2 cm TIM thickness, the absorber plate temperature has varied only from 68 to 70 °C; because most of the heat flux is dissipated to the side and top wall of the dryer due to less thermal storage capability. Similarly, in the solar dryer with no TIM storage, the maximum absorber plate temperature was observed to be 65 °C. From the simulation results, it is ascertained that the optimum thickness of 4 cm TIM is efficient to valorize the algal biomass and lipids at higher temperature due to its better thermal storage. Generally, the performance of the solar dryer depends on the collector temperature, absorber temperature, air temperature, and TIM. The solar dryer without TIM can dissipate its absorber plate temperature to the environment during poor weather, and night time and thus, it may affect the drying of biological samples by taking a long time. To overcome this challenge, the solar dryer was fabricated with cost-effective glass wool (TIM) as thermal storage medium and tested for valorizing biomass and lipids of the microalga *C. vulgaris*. The biomass of the *C. vulgaris* strain dried under BTPSD with respect to drying time (h) was presented in Fig. 2. The initial wet weight of the sample was taken as 1.0 g. The biomass weight was reduced gradually with an increase in drying time due to the evaporation of the water. It is apparent that at 3 and 4 h drying time, the wet algal biomass has been completely dried in BTPSD and the final dry weight of the sample was estimated to be 0.18 g, whereas partial drying was noticed at 2 h drying time. From the results, it is known that 3 h drying time is sufficient to valorize/evaporate the maximum moisture content in the microalgal wet biomass using BTPSD.

**Figure 3:**
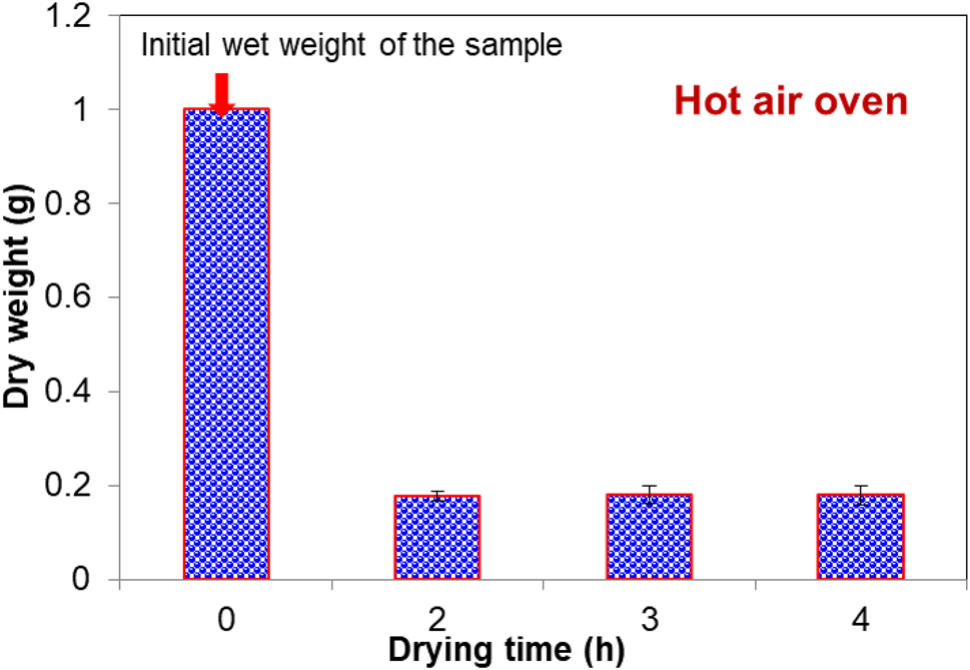
The conventional hot air oven based drying of algal biomass with respect to time (h).

On the other hand, the conventional hot air oven based drying of algal biomass was presented in Fig. 3. Like BTPSD, the same weight of wet biomass (1.0 g) was taken to study the weight loss with respect to drying time using Hot air oven. Unlike BTPSD, hot air oven has taken less time to dry the algal biomass, i.e., 2 h. The complete drying or complete removal of moisture was noticed at 2 h, and extending the drying up to 4 h did not reveal any remarkable change in the biomass weight due to no additional moisture removal and the final dry cell weight (DCW) of the taken sample was 0.18 g. Comparing both drying systems, the time taken for drying the algal sample by BTPSD and hot air oven is quite different. Though BTPSD requires an additionally 1 h for drying the sample compared to a hot air oven, the energy consumption and cost intensiveness are low in BTPSD.

**Figure 4:**
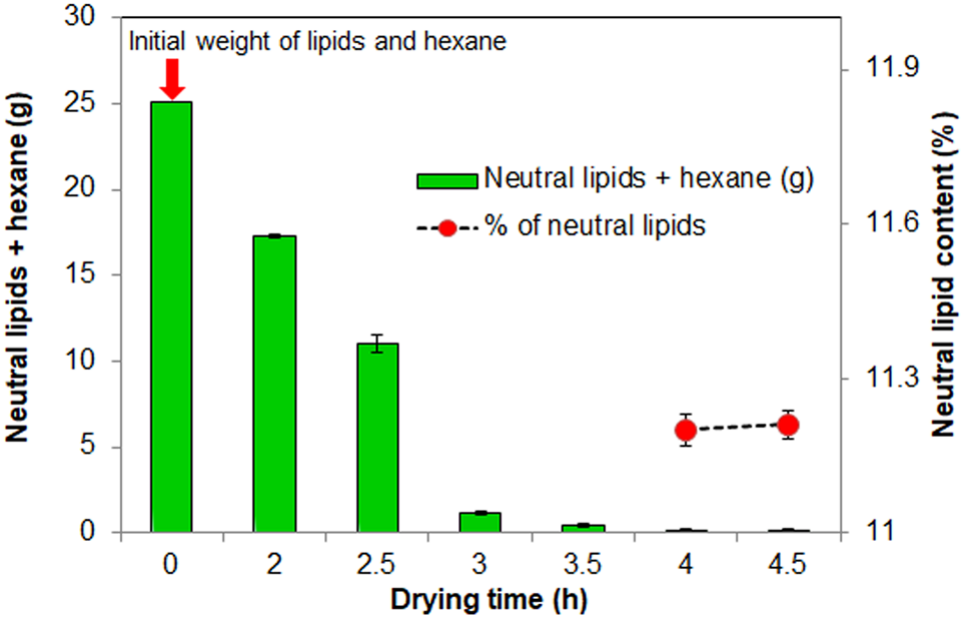
BTPSD based drying of hexane containing neutral lipids extracted from *C. vulgaris* strain.

**Figure 5:**
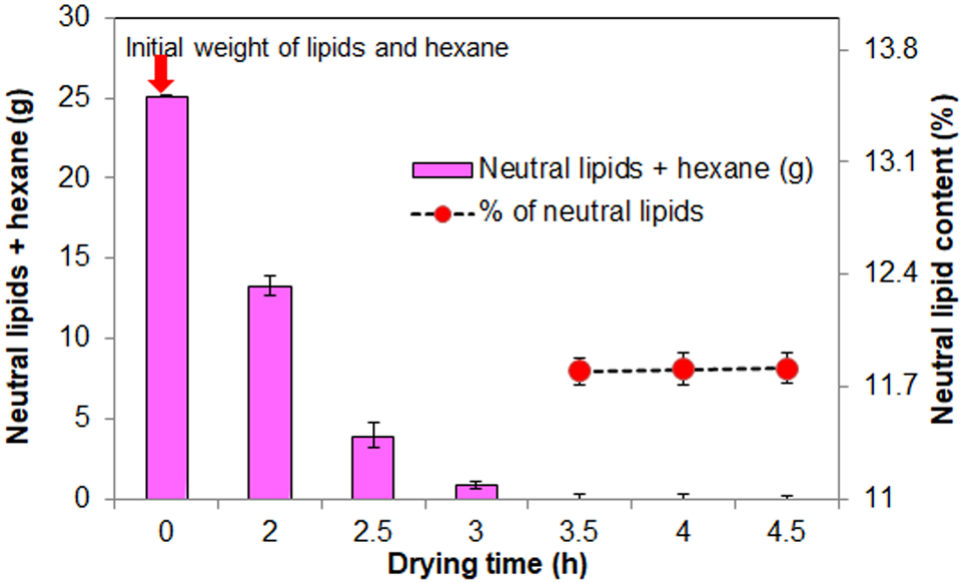
Hot air oven based drying of hexane containing neutral lipids extracted from *C. vulgaris* strain.

**Figure 6:**
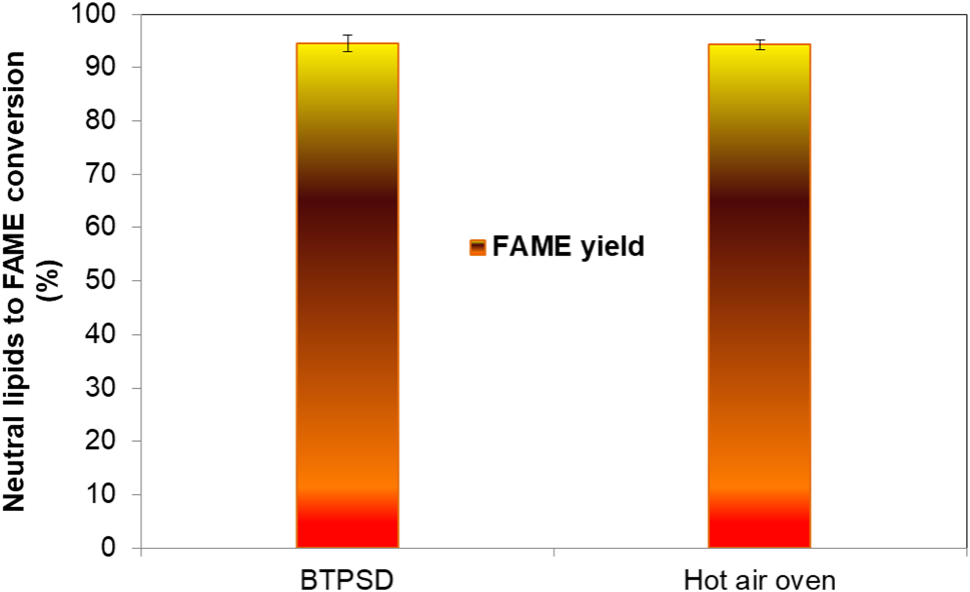
The FAME yield of *C. vulgaris* neutral lipids dried under BTPSD and hot air oven.

**Figure 7:**
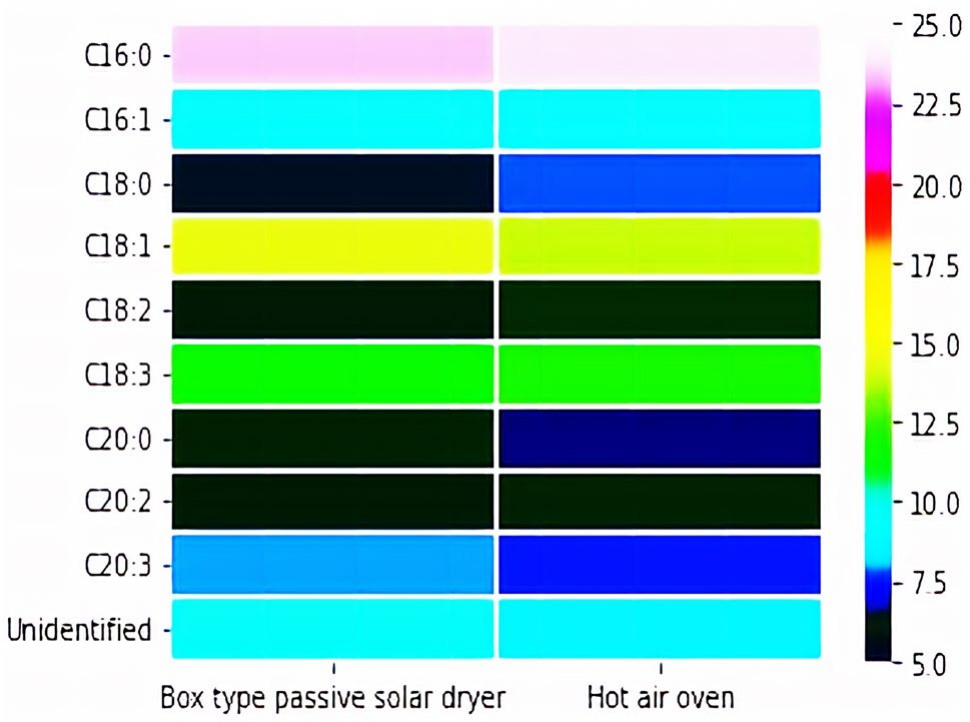
Heat map plotting of fold change ratio of fatty acids under BTPSD and hot air oven system.

Further, it is interesting to note that the final weight of the dried sample from both methods was found to be the same at about 0.181 g DCW. This is attributed to the fact that 4 cm TIM is able to increase the absorber plate temperature above 65 °C in BTPSD, which will evaporate the water content in the biomass. Though the drying time taken by BTPSD is high compared to hot air oven, low energy usage and less capital investment made the BTPSD a frontline drying system than a hot air oven. The dried biomasses from BTPSD and hot air oven were subjected to neutral lipid extraction using hexane. The hexane layer containing neutral lipids was kept in both drying systems for evaporating solvent (hexane) from neutral lipids. The BTPSD based drying of hexane-containing neutral lipids extracted from *C. vulgaris* strain was given in Fig. 4, with respect to drying time (h). The initial weight of the hexane containing non-polar lipid/neutral lipid was 25.13 g. The evaporation of solvent from the lipids were noted at 0.5 h time interval from 0 to 4.5 h; results indicated that hexane in the neutral lipids were completely evaporated at 4 h drying time and extending the duration up to 4.5 h, no further reduction in lipid weight/ no further evaporation of hexane was observed. Therefore, 4 h drying time is adequate to evaporate the hexane from the neutral lipids in BTPSD. Meanwhile, the neutral lipid percentage of *C. vulgaris* dried under BTPSD was calculated and it was 11.5%. As a comparison, a similar weight of hexane containing neutral lipids extracted from *C. vulgaris* was also taken in Hot air oven to evaluate the drying time difference between BTPSD and hot air oven. Figure 5 illustrates the hot air oven-based drying of hexane containing neutral lipids extracted from *C. vulgaris*. Unlike BTPSD, hot air oven has evaporated the solvent in less time at 3.5 h, i.e., 0.5 h earlier than BTPSD. In hot air oven, evaporation of solvent from the neutral lipids was occurred gradually from 0th h to 3.5 h and extending the drying duration beyond 3.5 h did not result any variation in the neutral lipid content i.e., neutral lipid percentage of 3.5 h, 4 h and 4.5 h drying time was 11.80, 11.81 and 11.81%, respectively. Based on the results, it is observed that both in BTPSD and hot air oven drying, the percentage of neutral lipids did not vary  concerning  the drying time and drying methods. Though the drying time is somewhat lesser in a hot air oven than in BTPSD, the energy consumption or expensiveness of the hot air oven process is very high. Notably, the 0.5 h drying time difference between BTPSD and hot air oven is not remarkably big. Hot air oven-based drying is a mechanical method in which hot air is supplied to remove the moisture at set temperature and velocity and it is considered to be aseptic method and yields the dried product with enhanced uniformity^7^. But, BTPSD based valorization of biomass and lipid is advantageous in terms of energy consumption and cost intensiveness than hot air oven. Further, BTPSD fabricated in this study is cost-effective, aseptic, eco-friendly as the entire setup was made with transparent glass materials without electrical power supply. Glass plate has low convective and radiant heat loss from the absorber plate and also it transmits the incident solar radiation to the receiver plate with less heat dissipation with the environment. Different types of solar dryers are designed and fabricated by various researchers to remove the moisture content from different foods and agricultural products. Various studies used solar dryers for drying fruits and agricultural products such as mango, banana, pineapple, cherry and potato, etc.^10^. In direct/open solar dryer, the quality of end product may not be maintained for human consumption and also, slow drying time may cause degradation of biomass and subsequent increase in bacterial population due to low temperature^15^. That is why, in this study, a closed box-type solar dryer was fabricated to avoid the contamination of sample from air-borne microbes and also to attain high temperature by reducing much heat dissipation. A closed type dryer increases the rate of drying and it results in high quality dried biomass, and also, the cost of drying algal biomass is relatively very less when compared to other drying methods^15^. Solar dryer are basically classified into active and passive solar dryer according to the nature of air flow and mode of incident solar radiation. In an active/forced air drying system, forced air is supplied to the dryer through external collector channel. But, in this study, passive type solar dryer was fabricated, in which, the solar radiation is collected by the transparent glass plate and then absorbed by the receiver plate without any external air supply. Solar dryers have many advantages like inexpensiveness, emission free, lesser spoilage, hygienic and eco-friendly^12^. One significant limitation is that this system can be used only during day-time (Sunshine hours) and in this case, a thermal backup is necessary to operate the dryer during night time^17^. Many thermal storage materials are being used, including natural sources like wood, stones, concrete, sandstone, bricks, etc. However, they can be suited for sensible heat of energy storage with low and high heat storage application^18^. For better thermal backup, some advance phase change materials are preferred as thermal insulations, namely water, salt hydrates, paraffins, selected hydrocarbons, polymers and metal alloys. PCMs maintain the inside heat of the dryer according to their specific heat capacity of the thermal insulation^19^. Phase change material is used to absorb excess energy during sunshine hours and then discharge the same at night/evening hours to enhance the drying^20,21^. In this study, glass wool was used as thermal insulation material (TIM) in the BTPSD. The performance of the BTPSD and hot air oven was given in the Table 1. The moisture content and moisture removal percentage of both systems were similar, i.e., 82 and 81.8%, respectively. The total energy required and total electricity consumed by hot air oven to remove the moisture content from the algal biomass was 138 J/g and 0.0385 Wh, respectively while the electricity consumption and total energy required by solar dryer was zero. Interestingly, capital investment/manufacturing cost of the BTPSD system is also meager when compared to the hot air oven. In solar dryer, removing the moisture or solvents from the samples requires hot air, which will be supported in two ways; (i) natural convection- does not require any other subsystem and energy consumption is very less, (ii) forced convection- requires a specific air compressor to supply the air to the drying chamber and it increases the system complexity and results in higher energy consumption^22^. Further, entire design of solar dyer was classified into three types (a) direct solar dryer (b) DC heating Dryer (c) PV/T based solar dryer^23^. Of all the models described above, direct, box-type solar dryer was simple in fabrication and was found to have economic feasibility^24^. The neutral lipids dried under hot air oven and BTPSD was converted into biodiesel (FAME) through acid-catalyzed transesterification. The FAME content obtained from the neutral lipids dried under BTPSD and hot air oven was presented in Fig. 6. FAME yield under BTPSD and hot air oven methods were 94.5 and 94.2%, respectively. As the percentage of neutral lipids content in both conditions was similar, FAME percentage was also found to be tantamount. Further, the fatty acid profile of *C. vulgaris* lipids dried under BTPSD and hot air oven was presented in Table 2. Additionally, the heat map plotting of the fold change ratio of fatty acids under BTPSD and the hot air oven system was given in Fig. 7. The key fatty acids present in the *C. vulgaris* strain are palmitic acid (C16:0), palmitoleic acid (C16:1), stearic acid (C18:0), oleic acid (C18:1), linoleic acid (C18:2), linolenic acid (C18:3), arachidic acid (C20:0), cis-11, 14-Eicosadienoic acid (C20:2) and cis-8,11,14-Eicosatrienoic acid (C20:3). The fatty acid composition/pattern was almost similar in both drying methods. Adding to that, the percentage of each fatty acid was virtually identical in both BTPSD and hot oven dried lipid samples. Among the fatty acids identified, palmitic acid is a predominant fatty acid in the concentration of 23.4% and then, oleic acid (14.3%), linolenic acid (11.42%), palmitoleic acid (9.22%) in BTPSD. Similarly, hot air oven dried lipid sample showed 23.9, 13.9, 11.98 and 9.13% palmitic acid, oleic acid, linolenic acid and stearic acid content, respectively. The significant fatty acids identified by the present study in the *C. vulgaris* are well consistent with various *hitherto* reports. A recent study reported that *Chlorella vulgaris* yields C16:0, C18:1, C18:2 as major fatty acids in its total FAME composition^25^. Photoautotrophically cultivated unialgal cultures of *Chlorella vulgaris* contain 16 to 18 carbon long-chain fatty acids; notably palmitic acid and oleic acid^26^. Another study has reported *C. vulgaris* (strain UMT-M1) yields C16:0 and C18:1 fatty acids in the concentration of 36.9 and 31.3% (of total oil content), respectively^27^. Palmitic acid, palmitoleic acid, oleic acid, linoleic acid and linolenic acid are the key fatty acids in the laboratory grown *Chlorella* sp., at about 33.42, 15.42, 20.3, 10.96 and 12.01%, respectively^28^. To utilize the microalgal strain for biodiesel application, fatty acid composition and contents have to be evaluated since the chemical features of each fatty acid govern the fuel quality based on its chain length and saturation^29^. It is reported that the fatty acid composition and its structure decide cetane number, viscosity and heat of combustion, and these fuel properties are linearly correlated with fatty acid chain length^30^. In this study, cetane number might be in the desirable range since both methods SFAs contents such as palmitic and stearic acid contents are high. Generally, the cetane number of palmitic acid and stearic acid is 74.5 and 86.9^26^. Favourable cetane number range minimizes the white smoke formation and compression ignition delay^31^. In this study, fatty acid pool contains mixture of saturated fatty acids (SFAs), monounsaturated fatty acids (MUFAs) and polyunsaturated fatty acids (PUFAs). As shown in Table 2, percentage of SFAs, MUFAs and PUFAs in BTPSD method was 35.82, 23.52 and 31.18%, respectively while it was 36.69, 23.03 and 31.68%, respectively in hot air oven dried lipid sample. The presence of saturated fatty acids provides favorable cetane number for better oxidative stability of the biodiesel^32,33^, while higher levels of polyunsaturated fatty acids provides good cold flow properties to the biodiesel at low temperature since they have more than two double bonds^34,35^. Fuel properties of biodiesel is determined by the SFAs and UFAs ratio since SFAs will increase the longevity of biodiesel, while UFAs augments cold flow properties of biodiesel^36^. Interestingly, long chain MUFAs such as palmitoleic acid and oleic acid in this study are favorable since they impart both better cold flow characteristics and oxidative stability as they do not have *bis*-*allylic* carbon^37,38^. It is reported that the presence of monounsaturated oleic acid in biodiesel has become a compromise between cold flow properties and oxidative stability^26^. By and large, fatty acid composition and percentage of the neutral lipids dried under BTPSD is favorable for high quality biodiesel production.

**Table 1:** Performance of BTPSD and hot air oven.

Parameters	Hot air oven	BTPSD
Moisture content (%)	82	82
Moisture removed (%)	81.80	81.81
Time taken (h)	2	3
Total energy required (J/g)	138	–
Total electricity consumed (Wh)	0.0385	–
Capital investment (INR)	22,000.00	750.00

**Table 2:** Fatty acid profile of *C. vulgaris* dried under BTPSD and hot air oven.

Fatty acids	BTPSD	Hot air oven
C16:0	23.4	23.9
C16:1	9.22	9.13
C18:0	6.45	7.75
C18:1	14.3	13.9
C18:2	5.93	6.02
C18:3	11.42	11.98
C20:0	5.97	5.04
C20:2	5.87	6.12
C20:3	7.96	7.56
Unidentified	9.48	8.6
**SFAs**	**35.82**	**36.69**
**MUFAs**	**23.52**	**23.03**
**PUFAs**	**31.18**	**31.68**

Significant values are in bold.

## Materials and methods

### Algal strain and maintenance

Marine microalga *Chlorella vulgaris* (henceforth referred to as *C. vulgaris*) was used in this study. The unialgal and axenic cultures of *C. vulgaris* was inoculated in 100 mL Erlenmeyer flask containing ASN-III medium and maintained in thermostatically controlled culture room at 25 °C with 1500 lx white fluorescent light illumination under 12:12 h L/D photoperiod without any CO_2_ supply. Seed inoculum at late log phase were taken and used for scale up in 1000 mL Erlenmeyer flask using ASN III medium for further experiments.

### Harvesting of biomass for drying experiments

Briefly, *C. vulgaris* culture at stationary phase was centrifuged at 6000 rpm for 10 min, and the pellet was taken and supernatant was discarded. The pellet was added with distilled water, and again centrifuged at 6000 rpm for 10 min to remove any salt residues in the biomass pellet. The salt-free fresh biomass pellet was taken in a preweighed Petri dish and used for drying experiments in BTPSD and a hot air oven.

### Design and fabrication of BTPSD

**Figure 8:**
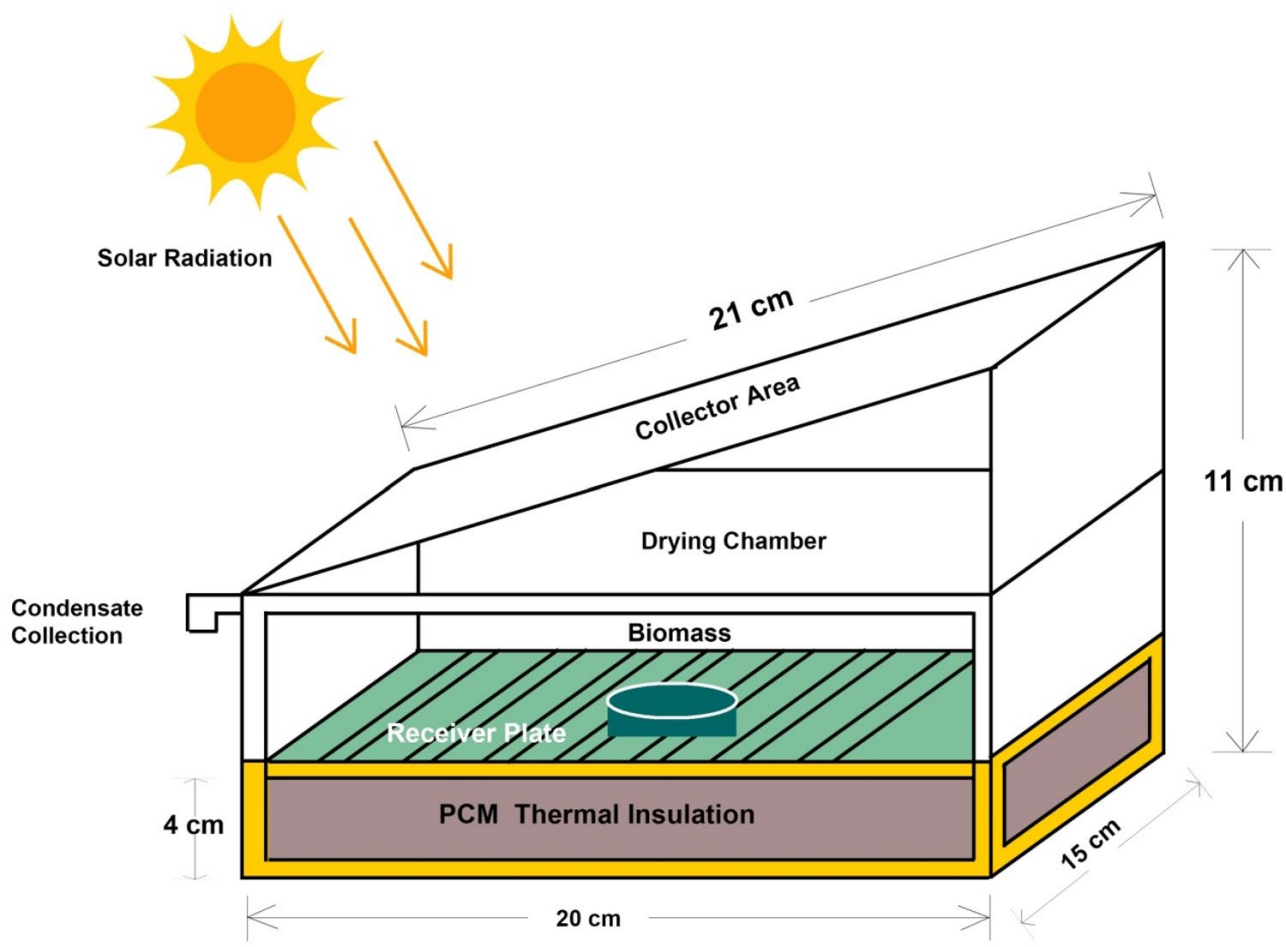
The schematic illustration of box-type passive solar dryer (BTPSD).

In the present study, box-type passive/direct type solar thermal dryer was designed and fabricated. A structural frame with the dimension of 20 cm × 15 cm × 11 cm (length × width × height) was designed for fabrication. The schematic diagram of BTPSD is shown in Fig. 8. The entire setup was fabricated with transparent glass materials, and the black copper plate was chosen as the receiver plate to enhance the heat absorption. The key limitation of the direct type solar dryer is thermal storage; but, in this proposed system, 4 cm thickness glass wool was used as TIM for thermal insulation (during poor weather or night time). In the proposed BTPSD, the solar radiation directly falls on the collector plate, and then, energy conversion occurs between the collector areas and receiver plate. The properties and condition of solar dryer is given in Table 3.

**Table 3:** BTPSD properties and condition.

Properties	Values
Wind speed	3–4.4 m/s
Ambient temperature	37 °C
Relative humidity outside	78%
Location of this study	Tiruchirappalli, India
Latitude	10.7589° N
Longitudes	78.8132° E
Facing the dryer	Due south
Dryer tilt	21 °
Specific heat capacity of TIM	2.14–2.9 J g^−1^ K^−1^

### Biomass drying in BTPSD and hot air oven

The known quantity of salt-free fresh biomass pellet (0.1 g) from “Harvesting of biomass for drying experiments” section was taken in a preweighed Petri dish and placed above the copper receiver plate of BTPSD. The solar dryer along with biomass was kept as due south facing with a 21 °tilt angle. Similarly, preweighed petri dish containing biomass pellet was placed in the hot air oven (Make: Labnet, Model: LTHOS-3). The time at which the experiments initiated was noted as 0 h for both BTPSD and hot air oven and the experiments were continued until the biomass completely dried (end time was noted). Then, dry cell weight (DCW) of *C. vulgaris* culture was valorized under BTPSD and hot air oven was calculated and expressed as g L^−1^ with response to time.

### Evaluation of solar dryer and oven performance

The performance of BTPSD with 4 cm TIM thickness was compared with the hot air oven system. The performance was evaluated based on the moisture removed, moisture content, the total energy required, and drying rate. The moisture content of the biomass depends on the relative humidity and temperature of the surrounding air.

i. *Moisture content (MC)* of biomass can be defined as water per unit mass of dry solids in the sample:
  $$\text{Moisture} \; \text{content }\left({\%}\right)=\frac{Initial \; weight \; of \; the \; sample-final \; weight \; of \; the \; sample}{Initial \; weight \; of \; the \;sample}*100$$
ii. *Moisture removed* (Mr) from the biomass product was calculated using the following equations,
  $$Moisture \; removed=m_{p}\frac{M_{i}-M_{f}}{100-M_{f})}$$
  where, m_p_—Initial mass of biomass (g), M_i_ and M_f_ are initial moisture content and final moisture content respectively (in %).
iii. *The total energy* required to evaporate the water content from the sample
  $${\text{E }} = {\text{ m}}_{{\text{p}}} {\text{C}}\Delta {\text{T}}$$
  m_p_—initial mass of biomass, C—specific heat of the water (4.2 J/kg C^−1^), ΔT—initial and final temperature difference

### Lipid extraction and estimation

Dried biomasses (BTPSD and hot air oven) from “Biomass drying in BTPSD and hot air oven” section were taken in the round bottom flask for neutral lipid extraction and estimation. The single solvent system comprising hexane was added to the biomass and allowed to reflux for 2 h at 80 °C with a water condenser^39,40^. At the end of reaction, crude lipids along with solvent were taken in separatory funnel and water was added for phase separation and the top solvent layer containing lipids were collected and kept for drying in both BTPSD and hot air oven. Eventually, solvent-free neutral lipids from both drying conditions were estimated at %.

### Biodiesel production through acid catalysed transesterification

Neutral lipids of *C. vulgaris* were converted into biodiesel through acid catalysis^32^. To begin with, known amount of neutral lipid was added with 3% sulfuric acid dissolved in methanol and allowed for reflux at 70 °C for about 2 h. At the end, hexane was added to the crude mixture for phase separation and the top hexane layer comprising FAME (fatty acid methyl ester) was taken and washed with water to get rid of water-soluble impurities. Water washing of FAME was continued until lower water layer reaches the pH 7. Then, top hexane containing purified FAME layer was taken for drying, and eventually, the FAME percentage was calculated.

### Fatty acid composition analysis of biodiesel by gas chromatography

FAMEs were evaluated for their fatty acid composition through Gas chromatography (Make: Shimadzu, Japan) equipped with a flame ionization detector using FAMEWAX Column. Detector temperature; 260 °C, Final oven temperature; 230 °C, Carrier gas; Nitrogen, Split ratio; 10:1 and Fatty acid standard used; Supelco 37 FAME mix. At the end of run, retention time of each fatty acid was compared with standard for the identification and quantification in the overall fatty acid.

### Statistical analysis

The BTPSD model was simulated through ANSYS FLUENT 19.2 Computational Fluid Dynamics tool to acquire its thermal characteristics. The entire design and experimentation are selected as per the geometrical condition of NIT, Tiruchirappalli, India. The thermal performance of the dryer is increased by using different TIM thicknesses. For simulation, BTPSD with 2 cm and 4 cm TIM thickness and BTPSD with no TIM were chosen and input temperature of 50 °C was fed into the software to examine the temperature profile of the BTPSD. All the experiments related to biomass and neutral lipids drying by BTPSD and hot air oven were carried out in triplicates and standard errors were indicated on the Figures.

## Conclusion

The cost-effective production of microalgal biodiesel involves the development of many lucrative methods and less energy-consuming downstream biomass processing. However, dewatering using mechanical methods consumes a lot of energy and is also expensive. Therefore, drying of algal biomass and lipids in a cost-effective way may further bring down the overall outlay of microalgal biodiesel production. Hence, in this study, box type passive solar dryer was fabricated and used to valorize algal biomass and neutral lipids compared to a hot air oven drying. The entire solar dryer was fabricated with glass wool as thermal insulation material for zero energy consumption, and thermal analysis was simulated using ANSYS FLUENT 19.2 Computational Fluid Dynamics tool. In this study, though the time taken for drying biomass and neutral lipids of *C. vulgaris* by solar dryer is slightly longer than hot air oven, the quality, quantity of biomass, neutral lipids and fatty acid pattern in both BTPSD and hot air oven dried samples was similar. Therefore, the present study’s results put forward that box-type passive solar dryer can be a promising, cost-effective system to valorize *C. vulgaris* biomass and neutral lipids for the industrial-scale production of biodiesel in the near future. Further studies are planned to operate the solar dryer with photovoltaic input in large-scale dewatering of biomass and also to calculate the evaporated solvent volume from the high volume of lipids drying through the condensate collector.
